# Dental Journalism

**Published:** 1881-03

**Authors:** 


DENTAL JOURNALISM.—We know of but one Dental Journal in this country, which is not published by a Dental Depot, for the purpose of advertising and selling dental goods. Should this be? Many of these periodicals are sent *gratis* to dentists for the benefit of the publishers. Others give premiums to paying subscribers. Does it take so much encouragement to induce those practicing the specialty of dentistry to read? They, as a class, are more able to pay for scientific literature than physicians. Their time which they might devote to reading is not so limited. The physician is more liberal, and he expects to pay for the journals he reads. Doubtless the advertising depot journals have taught the dentist to be less appreciative of dental literature.

Dentists write but little. This new specialty presents a broad field for thought and work. We receive letters from dentists all over the country saying that they will give us an article for our journal, when they can find time to write, with the spirit sweetly whispering in their ear. My brother, have you seated yourself a quiet evening with your fragrant Havana, (if you are so unfortunate as to be a smoker,) and invited that gentle angle to help your thoughts to flow through your pen for the benefit of the readers of THE INDEPENDENT PRACTITIONER? You say it is hard to write for a journal like ours, read by the general scientists, and those practicing all the specialties of medicine. Do not hesitate but give us a *pure dental* article, it may be from your laboratory or operating room. Do not soar up into the third heavens and use a multitude of silvery words to tell us that John has the tooth-ache, but write in a plain and concise style. The scientist will read it with the same benefit and interest that you derive from his articles. Your experience and teachings may lift a heavy load from many despondent brothers.

By the commingling of medicine and dentistry in our Journal we hope to place the latter where it of right belongs, i. e.: a specialty of the former. This we can not do alone, but come between and introduce to you like topics of mutual interest. "Each demands the aid of the other, and conspires amicably to the same end." The Journal is supported by its subscribers and advertisers, all of whom pay, or have promised to do so. Our work, so far, has been "a labor of love," and we trust with your encouragement and help, my brother Dentist, to give you a journal in which the Dentist, Surgeon, Occulist, Obstetrician, Physician, Scientist, &c., will meet on the same level. This is a work of time, and cannot be done without your co-operation. Will you help us in our *independent* journal, in which each can express freely his opinions, and read those of others in all the departments found in our pages? The INDEPENDENT PRACTITIONER is not purely a dental journal, and for that reason we feel that all dentists who hope to elevate themselves, and thereby raise the standard of their specialty, should aid us in our feeble efforts. We do not mean to speak disparagingly of dental journals—they have done a noble work.

It is a mistake to suppose that a well written and practical dental article would be unacceptable to physicians. Those gentlemen are called upon to practice in all the specialties of the medical profession, and dentistry, should be understood in order that cases requiring aid and diagnosis in that department may receive proper attention from the medical practitioner, particularly, in communities where there are no dentists. Physicians as such, leave the medical schools, with merely a general knowledge of dentistry, and none of them object to information, dental or otherwise, which is calculated to benefit or relieve their patients.

These remarks are added to the above article, by our junior, in order to urge intelligent dentists to contribute to our pages. We welcome well written articles from specialists, as well as the general practitioner, as it is our wish to make the Journal a useful and welcome visitor to all our friends.

